# A Liver-First Approach Using Ablation for Oligometastatic Pancreatic Cancer: A Report of a Successful Case

**DOI:** 10.7759/cureus.46671

**Published:** 2023-10-08

**Authors:** Shinichi Ikuta, Takayoshi Nakajima, Tsukasa Aihara, Masataka Fujikawa, Naoki Yamanaka

**Affiliations:** 1 Department of Surgery, Meiwa Hospital, Hyogo, JPN

**Keywords:** conversion surgery, ablation therapy, liver-first, liver oligometastases, pancreatic cancer

## Abstract

Pancreatic cancer (PC) is a highly aggressive malignancy, often accompanied by liver metastases as a common manifestation. While palliative chemotherapy remains the mainstay treatment for liver metastatic PC, local treatment approaches have gained attention, especially for patients with oligometastasis who exhibit a positive response to chemotherapy. This case report illustrates the successful application of a liver-first strategy in a 79-year-old male diagnosed with liver oligometastatic PC, originating in the pancreatic tail. The strategy encompassed percutaneous microwave ablation for liver metastases, followed by FOLFIRINOX (5-fluorouracil, folic acid, irinotecan, and oxaliplatin) chemotherapy, and subsequent primary tumor resection. The patient has remained disease-free for over a year post-surgery. This multidisciplinary approach may hold promise for selected patients with liver oligometastatic PC, although further research and case studies are needed for comprehensive evaluation.

## Introduction

Pancreatic cancer (PC) is an extremely aggressive malignancy, with rapid systemic spread and a dismal prognosis, typically resulting in five-year survival rates of less than 10% [1]. Liver metastasis is common in PC, affecting over half of all cases, either at the time of initial diagnosis or developing over time [2,3]. The primary therapeutic approach for liver metastatic PC revolves around palliative chemotherapy [4].

In recent years, exploration of local treatment approaches, including surgical resection so-called “conversion surgery”, has gained attention, particularly for patients with oligometastasis who have achieved an objective response to chemotherapy [4,5]. However, due to the absence of conclusive results from well-designed prospective studies, current practice involves cautious patient selection based on individual considerations. In this report, we present a case of a patient with liver oligometastatic PC, who was successfully treated through a liver-first strategy. This approach involved initiating treatment with percutaneous ablation therapy for liver metastases, followed by systemic chemotherapy and ultimately primary tumor resection with pancreatectomy.

## Case presentation

**Figure 1:**
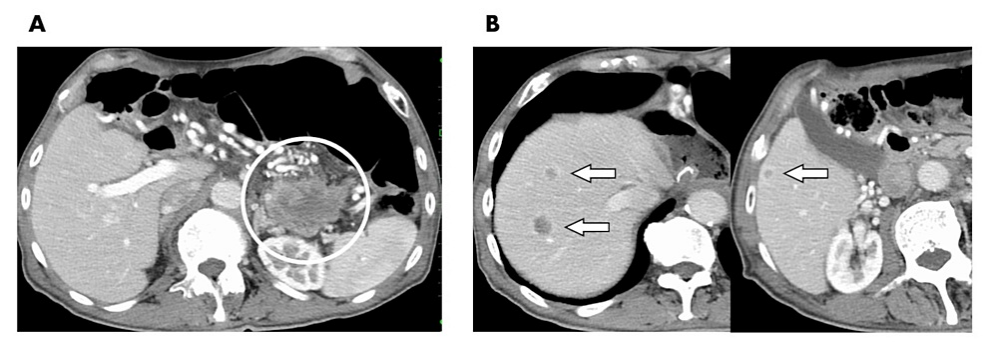
Pretreatment computed tomography images. (A) Pancreatic tail tumor (circle) and (B) metastases in liver segments 8 and 6 (arrows).

**Figure 2:**
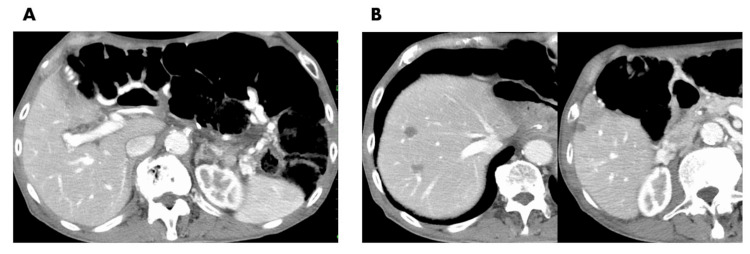
Computed tomography images after seven months of chemotherapy. (A) Shrunk pancreatic tumor and (B) shrunk coagulation area after microwave ablation with no evidence of local recurrence or emergence of new lesions.

A 79-year-old male presented with postprandial abdominal pain at an external hospital. He was diagnosed with pancreatic tail cancer through a computed tomography (CT) scan and an endoscopic ultrasound with fine-needle aspiration. No distant metastases were observed in the imaging diagnosis, which included positron emission tomography (PET) scans. Neoadjuvant chemotherapy, using gemcitabine plus nab-paclitaxel, was initiated with the aim of enhancing the prospects for a successful resection. However, two months later, liver metastases manifested, prompting a shift toward a non-resection strategy. The patient was then referred to our institution by his primary care physician to pursue further therapeutic interventions. A contrast-enhanced CT scan unveiled an irregular mass measuring 70 x 50 mm in the pancreatic tail, with occlusion of the splenic vein and development of collateral circulation (Figure 1A). Moreover, three metastatic tumors were identified in the liver: two in segment 8 with diameters of approximately 20 mm and 12 mm and one in segment 6 measuring 10 mm (Figure 1B). PET indicated no evidence of extra-hepatic metastases. The serum tumor markers, including carcinoembryonic antigen, carbohydrate antigen 19-9, and duke pancreatic monoclonal antigen type 2, did not exhibit a significant increase. After obtaining comprehensive informed consent, a multimodal treatment approach was developed, incorporating local treatment modalities. In the initial phase, ultrasound-guided percutaneous microwave ablation (The Emprint Ablation System with Thermosphere Technology, Covidien, Boulder, CO) was performed to address the three liver metastases. The ablation protocol primarily consisted of 75 W/0.5 min followed by 100 W/2 min, and two sessions of ablation were performed for the 20 mm tumor. After seven days of ablation therapy, the combination regimen FOLFIRINOX (5-fluorouracil, folic acid, irinotecan, and oxaliplatin) was introduced. Over 14 cycles of FOLFIRINOX spanning seven months, the primary tumor significantly reduced in size (Figure 2A). Effective control of liver metastases was achieved as well, with no signs of local recurrence or new lesions (Figure 2B). This ultimately led to a surgical procedure involving an open distal pancreatectomy, splenectomy, and the concomitant resection of the left adrenal gland, which was suspected of tumor involvement. Pathological analysis revealed adenocarcinoma with ypT1a, ypN0 (0/42), indicative of an Evans grade III response (>90% tumor cell destruction) [6]. Six weeks after the pancreatectomy, the patient resumed FOLFIRINOX treatment, receiving an additional 12 treatment cycles over a six-month duration. Currently, more than a year after surgery, the patient remains free from any signs of disease activity.

## Discussion

The liver-first approach has been a topic of discussion within the treatment strategy for colorectal cancer with synchronous liver metastases [7,8]. A key rationale behind this approach is to address the concern that liver metastases might become unresectable while treating the primary tumor, especially in cases of high liver tumor burden [7, 8]. Notably, if postoperative complications such as anastomotic leakage occur after resection of the colorectal tumor, there exists the possibility of subsequent treatment delays. However, whether a liver-first or primary-first approach is adopted, the risk of such treatment delays due to postoperative morbidities remains a persistent concern for patients undergoing planned staged surgery and chemotherapy.

Ablation therapy has gained widespread acceptance as a minimally invasive treatment option for various organs and tumor histologies. Compared to liver resection, ablation therapy offers advantages such as reduced morbidity, ease of repetition, and preservation of liver parenchyma [9]. While factors like large tumor size and difficult percutaneous access can limit its application, solutions could include choosing the appropriate ablation modality, utilizing artificial fluids, or employing laparoscopic techniques [10]. Limited reports have shown encouraging results for ablation therapy in treating liver metastases from PC [11,12]. Wang et al. demonstrated that radiofrequency ablation yielded improved tumor response (complete/partial response rate 72.22% vs. 27.78%), along with extended overall and progression-free survival, compared to systemic treatment in patients experiencing liver-only recurrence of PC post-surgery [11]. In a study of 104 patients with liver oligometastatic PC, Yan et al. reported that the combination of ablation and chemotherapy conferred notable survival advantages over chemotherapy alone [12].

In 2018, Lucchese reported an interesting case of PC with synchronous liver metastases that exhibited a favorable response to chemotherapy and underwent liver resection before the pancreatic resection [13]. However, this reverse approach could actually be referred to as "chemotherapy-first", as systemic chemotherapy preceded the liver surgery. Highlighted in our case is the potential benefit of adopting a liver-first strategy, utilizing ablation therapy, for the following reasons: (1) ablation therapy might surpass chemotherapy in controlling localized metastases and down-staging tumors, (2) reducing the risk of complications through a less invasive procedure, facilitating a seamless transition to chemotherapy, and (3) giving priority to ablation before chemotherapy can avoid the difficulty in applying local treatment for liver metastases, resulting from chemotherapy-induced tumor shrinkage, disappearance, or liver damage, associated with reduced visibility of the tumor in imaging studies.

Ultimately, provided that effective control of both metastatic and primary lesions can be sustained over a significant period of chemotherapy, pancreatectomy for the primary tumor becomes a viable option. While it is feasible to repeat ablation therapy for recurring liver metastases, the efficacy of additional local treatments might diminish, particularly in patients experiencing early tumor re-activation. As with the common conversion strategy, a careful assessment of tumor aggressiveness and chemoresponsiveness is indispensable to avoid futile pancreatectomy.

## Conclusions

To summarize, the liver-first strategy that incorporates ablation therapy shows potential as a treatment option for selected patients with liver oligometastatic PC. This approach allows for effective control of metastases without preventing a seamless transition to systemic chemotherapy, potentially enhancing the likelihood of subsequent primary tumor resection. While additional research and case studies are necessary to comprehensively assess its advantages, this multidisciplinary approach holds the promise of yielding improved outcomes for patients with liver oligometastatic PC.
